# Short-term fasting in glioma patients: analysis of diet diaries and metabolic parameters of the ERGO2 trial

**DOI:** 10.1007/s00394-021-02666-1

**Published:** 2021-09-06

**Authors:** Martin Voss, Katharina J. Wenger, Nina von Mettenheim, Jörg Bojunga, Manuela Vetter, Bianca Diehl, Kea Franz, Ruediger Gerlach, Michael W. Ronellenfitsch, Patrick N. Harter, Elke Hattingen, Joachim P. Steinbach, Claus Rödel, Johannes Rieger

**Affiliations:** 1 Dr. Senckenberg Institute of Neurooncology, University Hospital Frankfurt, Goethe University, Schleusenweg 2-16, 60528 Frankfurt/Main, Germany; 2 University Cancer Center Frankfurt (UCT), University Hospital Frankfurt, Goethe University, Theodor-Stern-Kai 7, 60590 Frankfurt/Main, Germany; 3 Partner Site Frankfurt/Mainz, German Cancer Research Center (DKFZ), German Cancer Consortium (DKTK), Stiftung Des Öffentlichen Rechts, Im Neuenheimer Feld 280, 69120 Heidelberg, Germany; 4 Frankfurt Cancer Institute (FCI), Georg-Speyer-Haus, Paul-Ehrlich-Straße 42-44, 60596 Frankfurt/Main, Germany; 5 Institute of Neuroradiology, University Hospital Frankfurt, Goethe University, Schleusenweg 2-16, 60528 Frankfurt/Main, Germany; 6 Department of Medicine 1, University Hospital Frankfurt, Goethe University, Theodor-Stern-Kai 7, 60590 Frankfurt/Main, Germany; 7 Department of Neurosurgery, University Hospital Frankfurt, Goethe University, Schleusenweg 2-16, 60528 Frankfurt/Main, Germany; 8 Department of Neurosurgery, HELIOS Hospital Erfurt, Nordhäuser Straße 74, 99089 Erfurt, Germany; 9 Institute of Neurology (Edinger-Institute), University Hospital Frankfurt, Goethe University, Heinrich-Hoffmann Strasse 7, 60528 Frankfurt/Main, Germany; 10 Department of Radiotherapy and Oncology, University Hospital Frankfurt, Goethe University, Theodor-Stern-Kai 7, 60590 Frankfurt/Main, Germany; 11 Interdisciplinary Division of Neuro-Oncology, University Hospital Tübingen, Hoppe-Seyler-Straße 3, 72076 Tübingen, Germany

**Keywords:** Glioblastoma, Radiation, Ketogenic diet, Fasting, Glucose, Leptin

## Abstract

**Purpose:**

The prospective, randomized ERGO2 trial investigated the effect of calorie-restricted ketogenic diet and intermittent fasting (KD-IF) on re-irradiation for recurrent brain tumors. The study did not meet its primary endpoint of improved progression-free survival in comparison to standard diet (SD). We here report the results of the quality of life/neurocognition and a detailed analysis of the diet diaries.

**Methods:**

50 patients were randomized 1:1 to re-irradiation combined with either SD or KD-IF. The KD-IF schedule included 3 days of ketogenic diet (KD: 21–23 kcal/kg/d, carbohydrate intake limited to 50 g/d), followed by 3 days of fasting and again 3 days of KD. Follow-up included examination of cognition, quality of life and serum samples.

**Results:**

The 20 patients who completed KD-IF met the prespecified goals for calorie and carbohydrate restriction. Substantial decreases in leptin and insulin and an increase in uric acid were observed. The SD group, of note, had a lower calorie intake than expected (21 kcal/kg/d instead of 30 kcal/kg/d). Neither quality of life nor cognition were affected by the diet. Low glucose emerged as a significant prognostic parameter in a best responder analysis.

**Conclusion:**

The strict caloric goals of the ERGO2 trial were tolerated well by patients with recurrent brain cancer. The short diet schedule led to significant metabolic changes with low glucose emerging as a candidate marker of better prognosis. The unexpected lower calorie intake of the control group complicates the interpretation of the results.

**Clinicaltrials.gov number**: NCT01754350; Registration: 21.12.2012.

**Supplementary Information:**

The online version contains supplementary material available at 10.1007/s00394-021-02666-1.

## Background

Malignant tumors are frequently characterized by higher glycolytic activity and glucose uptake [1, 2], which can be utilized diagnostically by positron-emission tomography using fluorodeoxyglucose as a tracer (FDG-PET) [3]. In brain tumors, increased glucose uptake correlates with poorer prognosis [4–6] and levels of serum glucose are a prognostic parameter in various tumor entities [7, 8] including brain cancer [9–11]. This led to the hypothesis that a diet with minimized carbohydrate intake, i.e., the ketogenic diet (KD) or modified Atkins diet, could selectively target the tumor vulnerability to glucose reduction. Isocaloric KD [12, 13] and especially calorie-restricted KD has shown meaningful activity in animal models of glioblastoma [14] supporting a dependency of glioblastoma cells on glucose and glutamine as their main energy source with potential impairment of mitochondrial energy generation and, therefore, reduced metabolic flexibility [15, 16]. In an in vivo animal study, Abdelwahab et al. reported a synergistic effect of radiation therapy in combination with KD. Nine out of 11 mice treated with KD and radiation therapy were apparently cured of their intracranial implanted tumor, as opposed to none of the animals in the radiation therapy only control group [17]. Retrospective analysis of the combination therapy showed that KD was feasible in human glioblastoma patients [18] and this concept has been proposed to be tested in a prospective clinical trial [19, 20]. We recently published results of the ERGO2 (Ernaehrungsumstellung bei Patienten mit Rezidiv eines Glioblastoms) trial. The trial was designed to analyze, whether a calorie-restricted KD could enhance the efficacy of re-irradiation in recurrent glioblastoma [21, 22]. We estimated that the addition of calorie restriction could increase the progression-free survival at six months (PFS6) to 30% in contrast to the expected 0% that had been published in this setting with radiation therapy [23].

Until now, the main mechanism of action and potential associated effects of a ketogenic diet in cancer treatment remain unclear. Isocaloric KD can reduce blood glucose to some extent and prevent reactive glucose/insulin increase as a deleterious side effect of some cancer treatments [24]. Yet isocaloric KD as a monotherapy approach has only very limited if any clinical anticancer activity. Additional fasting/calorie restriction reduces the interval needed to reach ketosis and increases the effect of KD [25–27]. Ketone bodies (acetoacetate and β-hydroxybutyrate) represent an alternative energy source for neurons during KD but may possess additional mechanisms of action as they decrease free radical damage, inhibit inflammation response and inhibit histone deacetylases (HDACs) [28–31].

Only seven other prospective clinical trials for KD in brain tumors have been reported thus far to our knowledge. Five trials were prospective, one-armed interventions [32–36] and only two trials were randomized using KD either in combination with perillyl alcohol [37] or in combination with first-line radiochemotherapy [38]. Reports of the dietary effects are often incomplete and do not allow a discussion of the mechanism of action of KD. We here report the pre-defined secondary endpoints of the ERGO2 trial with a detailed report of the quality of life, neurocognition, diet diaries and discuss the possible influence of the metabolic parameters on the trial’s negative results.

## Patients and methods

### Patients and treatment groups

**Fig. 1:**
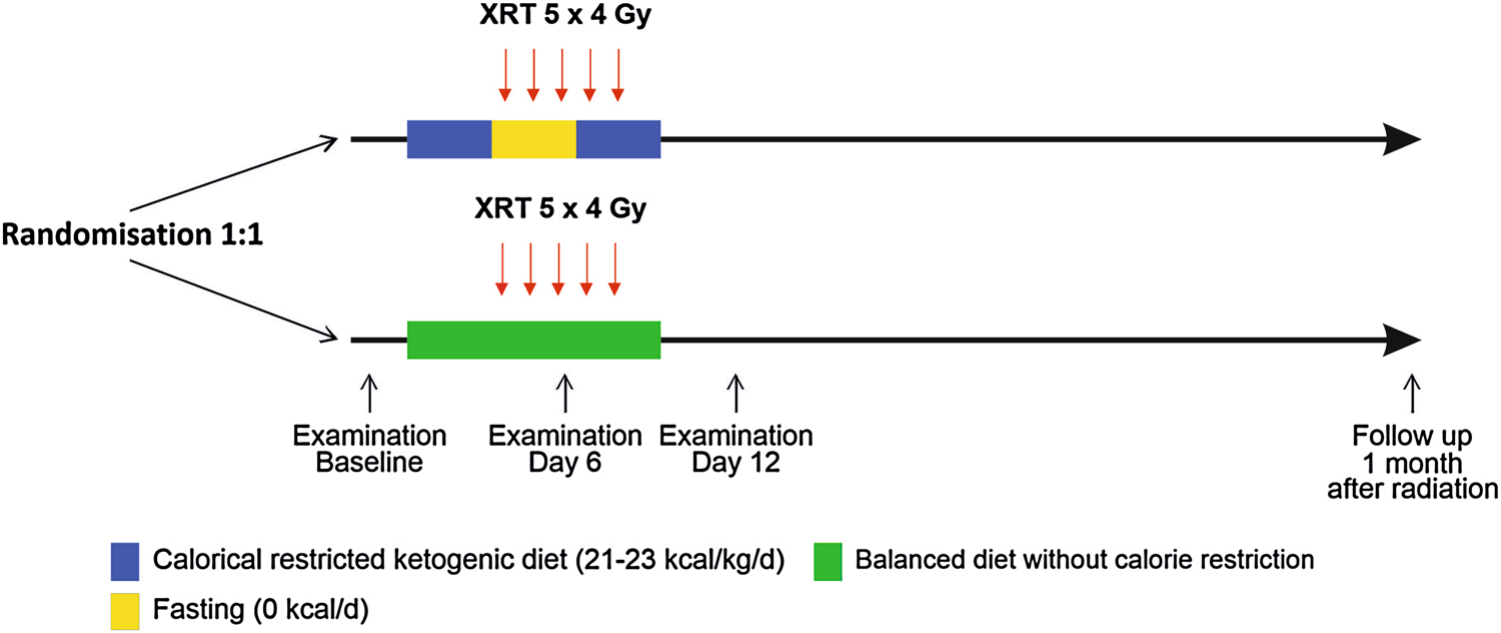
Schematic overview of the ERGO2 trial. The ketogenic diet/fasting group (KD-IF) started with a calorically restricted ketogenic diet on days 1–3. Patients fasted without calorie intake on days 4–6. Days 7–9 were again a calorically restricted ketogenic diet. Patients of the control group (SD) were counseled according to the recommendations of the German Society of Nutrition; no reduced calorie intake was desired. Radiation therapy (XRT) was planned from days 4 to 8 but final specification was left to the local radiation therapist. Figure adapted from [21]

The detailed description of the inclusion criteria and the consort diagram of the ERGO2 trial have been published previously [21]. In summary, 50 patients with recurrent glioblastoma were included and treated with re-irradiation. Patients were randomized 1:1 either to a standard diet (SD) according to the recommendations of the German Society of Nutrition (DGE) without calorie restriction or a combination of calorie-restricted ketogenic diet and intermittent fasting (KD-IF). Both groups received counseling from a professional dietician. The KD-IF intervention consisted of two calorically restricted KD intervals flanking 3 days of fasting (Fig. 1). A calorie restriction to 21–23 kcal/kg/d was intended and carbohydrate intake was limited to 50 g/d. The patients fasted on days 4–6 with unlimited intake of fluid. They were supplied with recipes for ketogenic meals and prefabricated ketogenic drinks were handed out as an alternative to the recipes or as a “rescue” meal in case of intense hunger (composition of the drinks in Supplement Table 1) which were kindly supplied by Tavarlin^®^ without costs. For patients in the standard diet (SD) group, a calorie intake of approximately 30 kcal/kg/d was expected. Patients with preexisting metabolic diseases like bowel obstruction, cachexia or insulin-dependent diabetes were excluded from the trial. The diet had to be terminated if a weight loss of more than 10% of the initial body weight, severe and symptomatic hypoglycemia or any WHO grade 4 toxicity possibly related to the diet occurred.

### Assessments

Patients were asked to fill out a dietary diary on days 1–12. At baseline, day 6, day 12 and 1 month after radiation therapy, patients were followed up by neurological examination, measurement of body weight, Karnofsky Performance Score, Mini-Mental-Status-Test, d2 test of attention and EORTC Quality of Life Questionnaire. At baseline, day 6, and day 12, blood samples were collected. Serum analyses of urea, uric acid, sodium, potassium, insulin and insulin-like growth factor 1 were performed as routine analysis in the hospital laboratory of the study site. Leptin was analyzed using the ELISA kit of DRG Instruments GmbH, Marburg, Germany.

### Quality of life

The Karnofsky Performance Status Scale (KPS) is a 100-point scale grading performance of activities of daily life as rated by the treating physician or study nurse [39].

EORTC Quality of Life Questionnaire (EORTC QLQ-C30, EORTC QLQ-Brain) was used for Quality of Life self-report by the patient. Raw scores were aggregated and transformed to a linear scale from 0 to 100 according to the algorithm described in the manual [40]. A higher score represents a higher level of functioning on function scales and a higher burden of symptoms on symptom scales. We selected six scales for the primary analyses: physical functioning (question 4), emotional function (question 23/24), social function (question 26/27), global health status (question 29/30), fatigue (question 18) and nausea/ loss of appetite (question 13/14).

### Neurocognition

The Mini-Mental State Examination (MMSE) was developed for detecting dementia in elderly people and assess orientation, memory, attention, recall, language and visual construction with a maximum total score of 30 points [40].

The d2 Test of Attention was used for neuropsychological evaluation of sustained attention ability [41, 42]. The test also focuses on visual scanning speed and can be hindered by impaired vision. The patients are asked to cross out any letter "d" with two marks around it on a sheet with distractors similar to the target stimulus, for example a "p" with two marks or a "d" with one or three marks. Primary data consist of totally processed letters and failures (missed target, wrong target crossed out) and result in a single value of attention ability (KL) which is standardized to the age group of the participant (KL-PR). The test is only validated in patients up to the age of 60 years. When the study subject was older than 60 years, standards table for 60-year-old subjects was used (13 of 42 patients treated by protocol).

### Statistical analysis

Microsoft Excel (Office 365) was used for data collection and overview. DGExpert software was used for data management of diet diaries and analysis of nutritional composition. SPSS Statistics Version 22 was used for statistical analysis. Independent samples *t* test was used for comparison between KD-IF and SD, while dependent samples *t* test was used for comparison between baseline and day 6. If normal distribution of serum values was not indicated by Shapiro–Wilk test (insulin day 6, IGF-1 baseline), Wilcoxon test was added to confirm the results. Chi-squared test used in the best responder analysis. PFS and OS were calculated by Kaplan–Meier survival analysis and log-rank test was used to compare survival times across treatment groups. Graphics were created in Corel Draw and Prism Graph.

## Results

### Update on PFS and OS

The first report on the ERGO2 trial reported no significant difference for progression-free survival (PFS6 as primary endpoint) and overall survival (OS) in the intention to treat cohort [21]. Data lock had been on January 2019. Analyzing the more mature data with data lock in April 2020, there was no change of the results. Mean PFS and OS still showed no significant difference (Supplement Fig. 1a and b). Unplanned subgroup analyses of the median split for glucose on day 6 (median glucose 83.5 mg/dl) had revealed a longer mean PFS for patients with glucose levels below the median in the KD-IF group. This too remained unchanged in the updated dataset (Supplement Fig. 1c).

### Quality of life and cognitive functioning

Health-related quality of life and neurocognitive functioning were evaluated during treatment and follow-up one month after radiation therapy for the intention to treat cohort. Further follow-up visits were confounded by tumor progression as 28 (56%) patients had a PFS of less than 100 days.

**Fig. 2:**
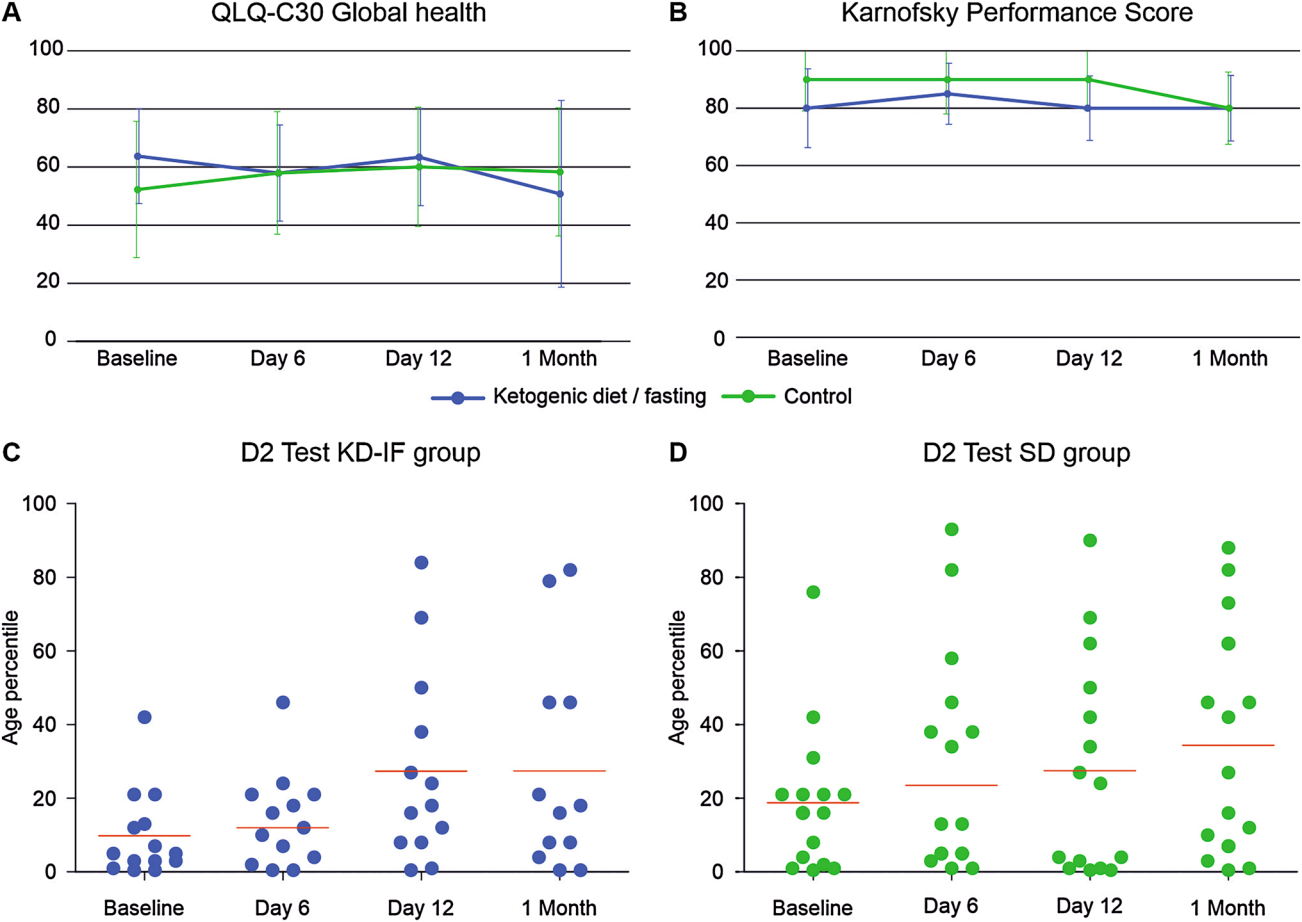
Quality of life and neurocognition. **A** Mean aggregated score for global health (Question 29/30) did not show a significant difference between the treatment groups or during the course of the trial. **B** There was no significant impairment in the activities of daily living estimated by KPS. **C**, **D** Each dot represents the individual result of a patient for the d2 test. Red bars indicate the median value. Baseline examination showed a severe impaired sustained attention ability of the patients on the age matched percentile for KD-IF (**C**) and SD (**D**). Test results improved significantly for both groups by repetitive testing during the follow-ups

Global indicator questions of the EORTC QLQ-C30 questionnaire revealed no significant difference between the two groups during treatment or at later follow-up (Fig. 2a). Data on physical, emotional and social function, fatigue and nausea/loss of appetite are displayed in the Supplement Fig. 2. Median KPS was 90% for SD and 80% for KD-IF with no significant change during the further assessment (Fig. 2b). There was no significant change in median MMSE as well (Median for KD-IF and SD at baseline and at follow-up after 1 month: 29 points).

Out of 42 patients who completed the intervention as set by the protocol, the d2 test was available for 29 patients (7 not done, 6 not possible) at baseline, for 28 patients at the end of the intervention at day 12 (7 not done, 7 not possible), and for 27 after one month (10 not done, 5 not possible). In contrast to MMSE, baseline evaluation of the d2 test revealed a severely impaired median cognitive function analyzing the age corrected percentile (KL-PR). Median KL-PR was 5% (range < 1–42% for KD-IF and 16% (range < 1–76%) for SD. Testing at day 6 revealed a non-significant increase of KL-PR in both groups with median KL-PR of 12% in the KD-IF group (*p* = 0.187) and 23.5% in SD group (*p* = 0.103) which increased significantly until follow-up after one month with median KL-PR of 17% in the KD-IF group (*p* = 0.035) and 27% in SD group (*p* = 0.049) (Fig. 2c, d).

### Metabolism

**Fig. 3:**
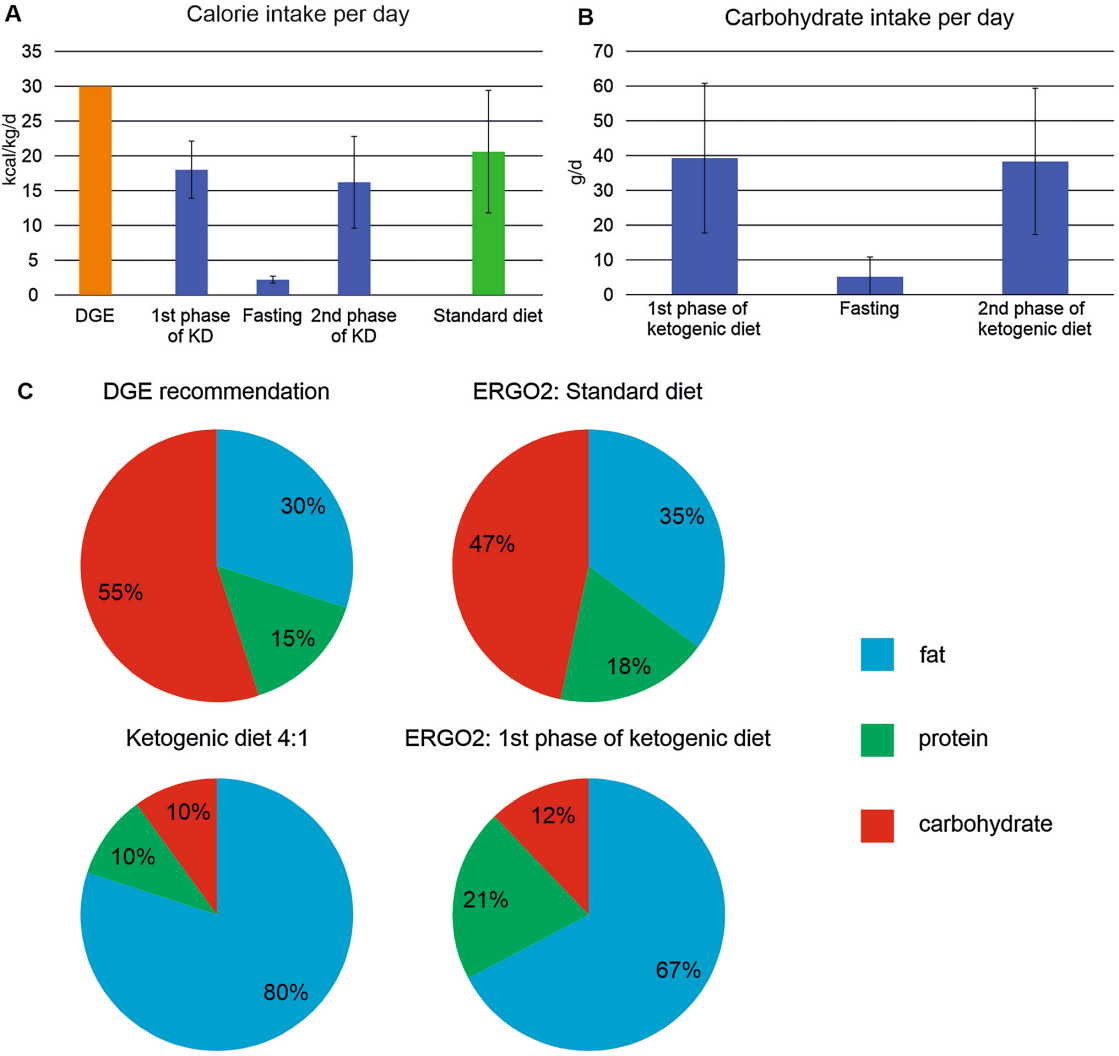
Diet composition. The analysis of the diet diaries of the per protocol treated patients. **A** The patients of the ketogenic diet/fasting group (KD-IF) group met the set goal of 21–23 kcal/kg/d with a mean intake of 18.0 ± 4.1 kcal/kg/d during the first three days and of 16.2 ± 6.6 kcal/kg/d during the second phase of calorie-restricted ketogenic diet. Calorie intake of the SD group was expected to be about 30 kcal/kg/d (German Society of Nutrition, DGE) but had been lower with an intake of 20.6 ± 8.8 kcal/kg/d. **B** The set carbohydrate restriction of at most 50 g/d could also be achieved by the KD-IF group with an intake of 39.3 ± 21.6 g/d and 38.3 ± 21.0 g/d in the phases of KD. (**C** upper row) The diet of the SD group matched the macronutrient composition recommended by the (DGE). (**C** lower row) Classic ketogenic diet without calorie restriction has a ratio of 3 or 4 g of fat to 1 g of protein and/or carbohydrate. Analysis of the days of ketogenic diet showed that a ratio of 2:1 could be achieved

The diet was accompanied by a diet diary that was later transferred to DGExpert software. Patients reported all their food intake and the number of ketogenic drinks consumed. Patients used a median of 12 drinks during the diet phase with three patients using only the drinks as ketogenic diet. An example for the detailed analysis of two patients is given in Supplement Table 2. A complete dataset of the diet was available in 17/20 (85%) per protocol treated KD-IF patients. The analysis of the diet diaries showed that patients could reach the set diet goals of 21–23 kcal/kg/d and the limit of 50 g/d carbohydrates during KD (Fig. 3a, b). Study patients had a mean intake of 1541 ± 310 kcal/d (18.0 ± 4.1 kcal/kg/d) during the first three days of calorie-restricted ketogenic diet and of 1320 ± 488 kcal/d (16.2 ± 6.6 kcal/kg/d) during the second phase of calorie-restricted ketogenic diet on days 7–9 of the protocol. Mean intake of carbohydrates during KD was 39.3 ± 21.6 g/d and 38.3 ± 21.0 g/d. During the three-day fasting period (days 4–6), calorie intake was lowered to a mean of 172 ± 253 kcal/d (2.2 ± 0.5 kcal/kg/d range: 0–790 kcal/d) and intake of carbohydrates was 5.1 ± 5.7 g/d (range 0–18 g/d). Classic ketogenic diet without calorie restriction has a ratio of 3 or 4 g of fat to 1 g of protein and/or carbohydrate. Analysis of the days of ketogenic diet showed that a ratio of 2:1 could be achieved (range 1:1–3.5:1) (Fig. 3c).

For patients in the standard diet (SD) group, nutrition counsel was given adhering to the recommendations of the DGE without advice for calorie restriction. The composition of the diet was close to the recommendation of DGE (Fig. 3c). Calorie intake of approximately 30 kcal/kg/d had been recommended which would correspond to a mean intake of 2312 kcal/d. The SD group had a lower intake of 1726 ± 612 kcal/d (20.6 ± 8.8 kcal/kg/d) despite or maybe because of the counseling (Fig. 3a). Intake of carbohydrates was 208 ± 79 g/d (range 86–491 g/d).

Because of the unexpectedly reduced calorie intake of the SD group, the difference between the 6 days of ketogenic diet (1st and 2nd phase of KD combined) and the average calorie intake of the SD group did not reach statistical significance (KD-IF ketogenic diet days: 17.1 ± 5.5 kcal/kg, SD: 20.6 ± 8.8 kcal/kg/d, *p* = 0.1). However, comparing the mean calorie intake of all 9 days, including the days of fasting in the KD-IF group, there was a large and statistically significant difference (KD-IF: 12.1 ± 8.6 kcal/kg, SD: 20.6 ± 8.8 kcal/kg/d, *p* < 0.01). The difference in mean carbohydrate intake was statistically significant when comparing the nine days of diet of KD-IF 28 ± 14 g/d to SD 208 ± 79 g/d (*p* < 0.01).

**Fig. 4:**
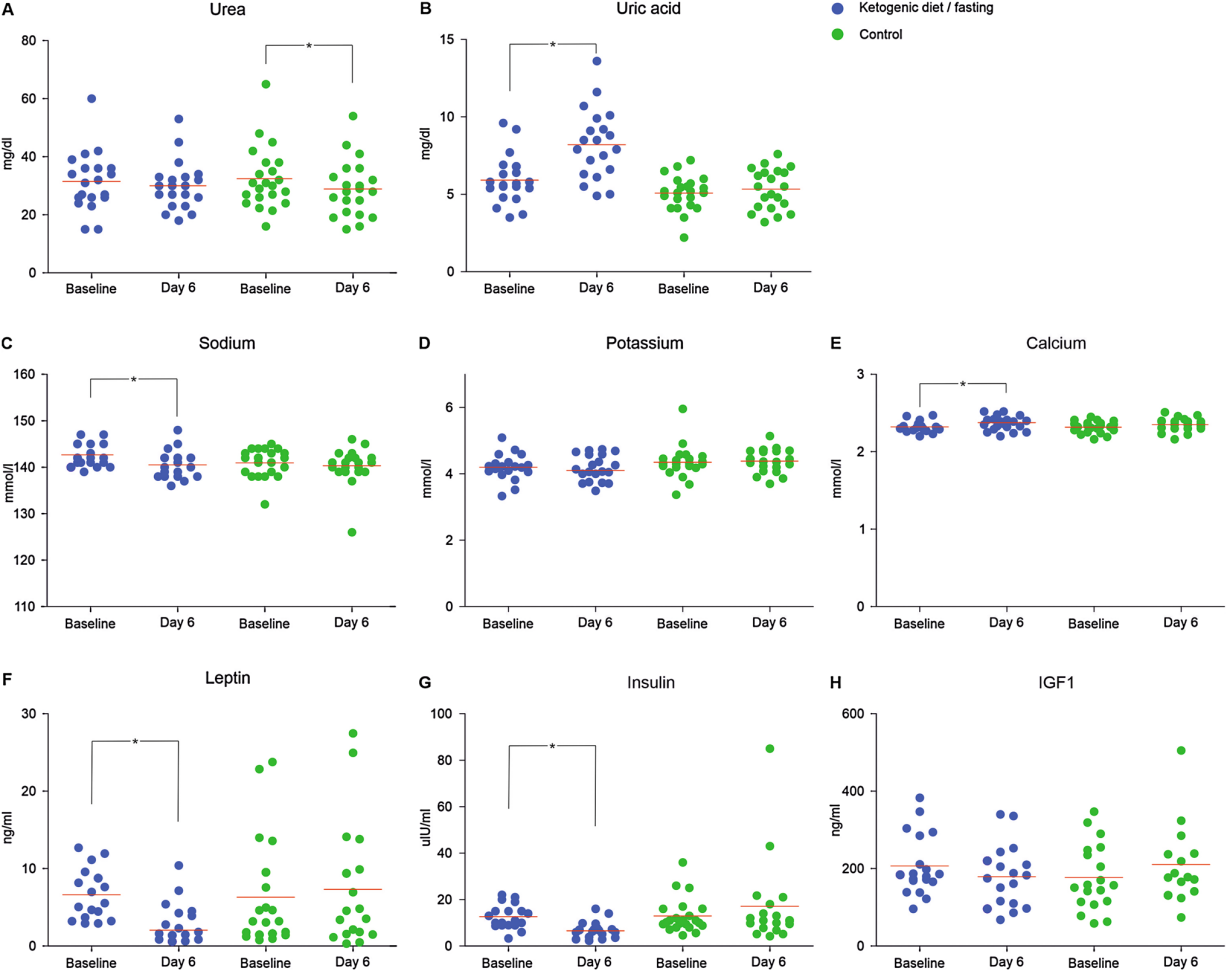
Serum analysis KD-IF Group. Each dot represents the individual result of a patient completing the intervention as set by the protocol. Red bars indicate the mean value. Statistically significant differences are indicated by *

Side effects of the ketogenic diet are well established during the first week of diet as the body adapts to the new composition of nutrients and is referred to as “keto-flu”. The gastrointestinal symptoms in addition to headache and muscle cramps are discussed to be the results of a loss of fluid and electrolytes as well as metabolic acidosis. Serum analysis of patients treated per protocol revealed no statistically significant changes in levels of urea (Baseline: 31.5 ± 10.1 mg/dl, day 6: 30.1 ± 8.4 mg/dl; *p* = 0.375), Insulin-like growth factor 1 (Baseline: 207.1 ± 78.3 ng/ml, day 6: 179.9 ± 79.7 ng/ml; *p* = 0.080) or potassium (Baseline: 4.2 ± 0.39 mmol/l, day 6: 4.2 ± 0.39 mmol/l; *p* = 0.824) from baseline to day 6. A significant increase of uric acid was noted in the KD-IF group (Baseline: 5.9 ± 1.6 mg/dl, day 6: 8.2 ± 2.3 mg/dl; *p* < 0.01) opposed to the SD group (*p* = 0.093) as well as an increase of calcium (Baseline: 2.32 ± 0.07 ng/ml, day 6: 2.38 ± 0.09 mmol/l; *p* = 0.031; SD *p* = 0.082). There was a significant decrease of leptin levels (Baseline: 6.6 ± 3.2 ng/ml, day 6: 3.1 ± 2.7 ng/ml; *p* < 0.01; SD *p* = 0.437), insulin (Baseline: 12.6 ± 5.2 µIU/ml, day 6: 6.9 ± 3.7 µIU/ml; *p* < 0.01; SD *p* = 0.261) and a small yet significant decrease in sodium (Baseline: 142.7 ± 2.4 mmol/l, day 6: 140.7 ± 2.9 mmol/l; *p* < 0.01; SD *p* = 0.460) (Fig. 4). Data on changes of glucose, ketone bodies, cholesterol (HDL, LDL) as well as triglyceride levels have been previously reported ([21] ERGO2 Supplement Table 1). Please note that the blood samples were drawn at day six, at the end of the three-day fasting period, and might reflect more the effect of the fasting than the effect of the prior ketogenic diet. Leptin and glucose levels at baseline in all patients had no significant correlation (*p* = 0.937). Neither had leptin and glucose levels at day 6 in the per protocol treated patients of the KD-IF group (*p* = 0.244). There was a trend towards statistical significance correlating ketone bodies and leptin at day 6 in the KD-IF group (*p* = 0.058).

Mean BMI was 27.3 ± 3.4 kg/m^2^ for KD-IF and 26.9 ± 4.3 kg/m^2^ for SD (*p* = 0.668) hinting at a generally overweight collective. Only 12 of the included patients had a normal weight (24%), 26 patients were classified as overweight/pre-obese (52%) and the remaining 12 patients were obese (24%). No underweight patients were included as this was an exclusion criterion. In an exploratory analysis, normal weight patients had the shortest PFS with a mean of 63 days (95%CI 38.9–87.8) compared to overweight patients with 146 days (95%CI 85.3–207.0, *p* < 0.01) and obese patients with 100 days (95%CI 51–148.9, *p* = 0.162) (Supplement Fig. 3). There was no statistically significant difference for OS (not shown). There was also no statistically significant difference in BMI between the 16 patients who were on corticosteroids at baseline compared to the other patients (*p* = 0.057). The weight loss during the trial is shown in Supplement Fig. 4.

### Analysis of best responders

To generate a hypothesis and to identify possible serum markers for future trials, the best responders of the KD-IF group treated per protocol were analyzed (20 patients). Only patients with primary glioblastoma histology were included (2 patients excluded) to warrant a homogeneous cohort. Best responders were defined as patients with a PFS greater than 100 days without any other specific statistical considerations while being aware of the analysis-by-responder bias (potentially identifying patients who had a better prognosis at baseline, guarantee-time bias). The profile of the best responders (Table 1) showed non-significantly better dietary response to the intervention with lower glucose, higher ketosis and lower calorie/carbohydrate intake during the fasting period. To be noted, the best responders had a higher rate of methylation of the MGMT promoter, no pre-treatment with bevacizumab and less treatment with dexamethasone.

**Table 1:** Best responders KD-IF group

	Best responder	Low responder	*p*
Number of patients	7	11	
MGMT promotor			
Methylated	4 (57%)	3 (27%)	0.152
Not methylated	2 (29%)	7 (64%)	
Not known	1 (14%)	1 (9%)	
Pretreatment bevacizumab			
No	7 (100%)	8 (73%)	0.130
Yes	0 (0%)	3 (27%)	
Glucose at baseline [mg/dl]	87.4 ± 15.6	87.3 ± 15.4	0.993
Bodyweight at baseline [kg]	82.7 ± 13.2	86.2 ± 10.2	0.532
Dexamethason at day 6			
No	5 (71%)	4 (36%)	0.147
Yes	2 (29%)	7 (64%)	
Glucose day 6 [mg/dl]	69.1 ± 10.8	81.6 ± 12.5	0.045*
Glucose relative to baseline	−18.3 ± 16.2	−5.7 ± 14.9	
Ketosis day 6 [mmol/l]	2.9 ± 1.6	1.6 ± 1.2	0.054
Leptin day 6 [ng/ml]	1.1 ± 10.8	3.5 ± 2.7	0.117
Leptin relative to baseline	−2.6 ± 0.5	−3.5 ± 3.6	
Change in bodyweight	−2.4 ± 2.0	−2.3 ± 1.9	0.983
Fasting calorie intake [kcal/d]	107 ± 202	238 ± 307	0.347
Fasting carbohydrate intake [g/d]	2.7 ± 3.1	7.3 ± 7.1	0.111
Progression-free survival [days]	261	50	< 0.01*
Overall survival [days]	485	250	0.030*

Asterisks represent statistically significant values (*p* < 0.05)

## Discussion

Despite an abundance of data of positive effects of calorie-restricted KD in glioma cells in preclinical models as well as synergistic effects with radiation therapy, the intervention in the ERGO2 trial failed to show a statistically significant difference in PFS between the treatment groups. After analyzing the dietary diaries, this was not due to the inability of patients to follow the strict calorie guidelines defined for the ERGO2 trial. In fact, patients succeeded in integrating the diet into their daily life and even exceeded the required calorie restriction during KD set at 21 kcal/kg/d with a median of only 17.1 ± 5.5 kcal/kg per day. The defined goals for carbohydrate intake and fasting were achieved similarly. A calorie intake of approximately 30 kcal/kg/d is recommended by the WHO and was the expected calorie intake of the control group. Nevertheless, analysis revealed that despite or perhaps because of the counseling, mean calorie intake of the SD group had been only at 21 kcal/kg/d per day. This most likely explains the failure to reach statistically significant difference with regard to calorie intake during the phases of KD between intervention and control group and could be an important confounding factor distorting the results of the trial. Comparing the cumulative calorie intake over the complete nine days of dietary intervention including the fasting period, we anticipated a reduction in the KD-IF group to 40% of calorie intake of the SD group. Yet the analysis revealed that the intervention had been less intense with a reduction to only 60% of the SD group due to the reduced calorie intake of the SD group. Additionally, it has to be pointed out that the composition of macronutrients during the phase of KD was in median only 2:1 instead of the desired 3–4:1 ratio.

Overall results of ERGO2 show that metabolic interventions targeting brain tumor metabolism need to be further refined. One hypothesis is, that the intervention was too 'weak' as it had been a mixed intervention of ketogenic diet and fasting. The days of relatively high calorie intake during ketogenic diet could have interfered with the well-established effect of fasting on IGF-1, glucose and insulin. In addition, nine days may have been too short to establish the optimal therapeutic window for synergistic effects with re-radiation therapy. These assumptions are backed up by the results of the MR-spectroscopic analysis of tumor and brain tissue performed at day 6 [22]. Ketone bodies (acetone) within tumor tissue and/or normal appearing white matter could only be detected in 4/11 patients and the intervention did not (yet) affect ATP levels. Instead of a single phase of diet intervention, repetitive cycles of fasting during the first-line therapy of glioblastoma might have a more pronounced effect on tumor metabolism.

Both the targeted analysis of reduction in glucose levels employed in the original ERGO2 report and the best responder analysis utilized here identify glucose as a candidate marker and possibly effector of fasting/KD interventions. For an incorporation of KD as a therapeutic approach in glioblastoma patients, it will be critical to define the required level and interval of serum glucose reduction to affect glioblastoma metabolism. Results of the best responder subgroup analysis suggest aiming for at least a slight hypoglycemia. In contrast, ketosis, leptin and weight loss were insufficient surrogate parameters for outcome. A correlation between serum glucose, ketones and other metabolic parameters and corresponding changes in the tumor tissue during fasting are the focus of the ERGO3 trial (NCT04461938) which is currently recruiting.

It is also possible that a dietary intervention like KD or fasting alone might not suffice to reduce blood glucose levels to a therapeutically relevant level which could require additional drug-based therapy. Such an approach might even allow employing a less strict dietary protocol. Metformin may be a primary candidate for this, since it has recently demonstrated clinical activity in the model brain tumors driven by the metabolic master regulator mTOR in tuberous sclerosis patients [43]. Synergistic effects may also be expected by combination with inhibitors of glycolysis like WP1122 [44] or by blocking glutamine metabolism [45].

In summary, the defined goals for calorie and carbohydrate restriction as well as the fasting period could be met by the patients with recurrent brain tumors without negative effects on the quality of life. The short diet schedule already led to significant metabolic changes suggesting that short-term dietary interventions might be therapeutically useful, possibly combined with other modalities. The unexpected lower calorie intake of the SD group might have hampered the interpretation of the trial.

## Supplementary Information

Below is the link to the electronic supplementary material.

Supplementary file1 (DOCX 4890 KB)

## Data Availability

Data were generated at the Dr. Senckenberg Institute of Neurooncology and are available from the corresponding author M.Vo. upon reasonable request.
