# Supplementary material for: Short-term fasting in glioma patients: analysis of diet diaries and metabolic parameters of the ERGO2 trial
Source: Eur J Nutr. 2021 Sep 6;61(1):477–87. doi: 10.1007/s00394-021-02666-1 (PMC8783850; doi:10.1007/s00394-021-02666-1)
Supplement: Supplementary file 1 — Supplementary file1 (DOCX 4890 KB) [file 394_2021_2666_MOESM1_ESM.docx]

**Supplementary Information**

**European Journal of Nutrition**

**Short-term fasting in glioma patients**

**– Analysis of diet diaries and metabolic parameters of the ERGO2 trial**

Martin Voss_1,2,3,4_, Katharina J. Wenger_2,3,4,5_, Nina von Mettenheim_1,2,3,4_, Jörg Bojunga_6_, Manuela Vetter_1,2,3,4_, Bianca Diehl_1,2,3,4_, Kea Franz_2,3,4,7_, Ruediger Gerlach_8_, Michael W. Ronellenfitsch_1,2,3,4_, Patrick N. Harter_2,3,4,9_, Elke Hattingen_2,3,4,5_, Joachim P. Steinbach_1,2,3,4_, Claus Rödel_2,3,4,10_ and Johannes Rieger_1,11_

_1_Dr. Senckenberg Institute of Neurooncology, University Hospital Frankfurt, Goethe University, Schleusenweg 2-16, 60528 Frankfurt/Main, Germany

_2_University Cancer Center Frankfurt (UCT), University Hospital Frankfurt, Goethe University, Theodor-Stern-Kai 7, 60590 Frankfurt/Main, Germany

_3_ German Cancer Consortium (DKTK), partner site Frankfurt/Mainz; and German Cancer Research Center (DKFZ), Stiftung des öffentlichen Rechts, Im Neuenheimer Feld 280, 69120 Heidelberg, Germany

_4_Frankfurt Cancer Institute (FCI), Georg-Speyer-Haus, Paul-Ehrlich-Straße 42-44, 60596 Frankfurt/Main, Germany

_5_Institute of Neuroradiology, University Hospital Frankfurt, Goethe University, Schleusenweg 2-16, 60528 Frankfurt/Main, Germany

_6_Department of Medicine 1, University Hospital Frankfurt, Goethe University, Theodor-Stern-Kai 7, 60590 Frankfurt/Main, Germany

_7_Department of Neurosurgery, University Hospital Frankfurt, Goethe University, Schleusenweg 2-16, 60528 Frankfurt/Main, Germany

_8_Department of Neurosurgery, HELIOS Hospital Erfurt, Nordhäuser Straße 74, 99089 Erfurt Germany

_9_Institute of Neurology (Edinger-Institute), University Hospital Frankfurt, Goethe University, Heinrich-Hoffmann Strasse 7, 60528 Frankfurt/Main, Germany

_10_Department of Radiotherapy and Oncology, University Hospital Frankfurt, Goethe University, Theodor-Stern-Kai 7, 60590 Frankfurt/Main, Germany

_11_Interdisciplinary Division of Neuro-Oncology, University Hospital Tübingen, Hoppe-Seyler-Straße 3, 72076 Tübingen, Germany

**Address for Correspondence**

Dr. Martin Voss

Dr. Senckenberg Institute of Neurooncology

Schleusenweg 2-16, 60528 Frankfurt am Main Germany

Mail: [martin.voss@kgu.de](mailto:martin.voss@kgu.de)

**Supplement Figure 1: Progression free survival and overall survival**


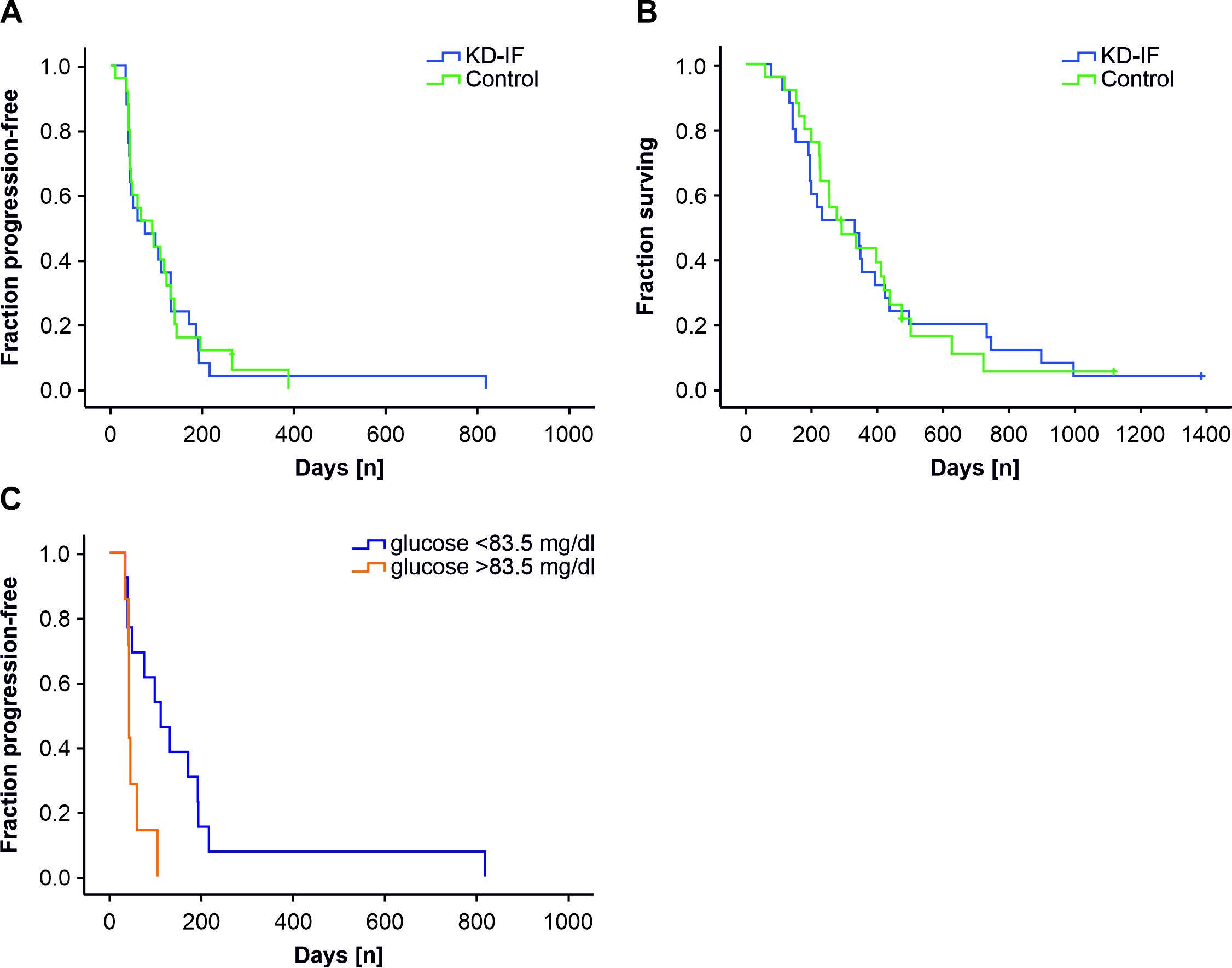


(A) Analysis of the intention to treat population (n = 50) revealed no significant difference for PFS with mean PFS of 122 days for KD-IF (95%CI 61–184) and PFS of 111 days for SD (95%CI 73–149) (p =0.845). (B) There was no difference for OS with mean OS of 394 days for KD-IF (95%CI 270-519) and OS of 374 days for SD (95%CI 274-473) (p =0.965). (C) Unplanned sub-analysis of the patients treated as set by protocol in the KD-IF group (n = 20). When comparing the patients with a glucose on day 6 (the last day of fasting) below the median of 83.5mg/dl to the patients above the median there was a statistically significant longer mean PFS of 167 days (95%CI 55-279) in comparison to 52 days (95%CI 34-70) (p =0.014).

**Supplement Figure 2: Health-related quality of life**


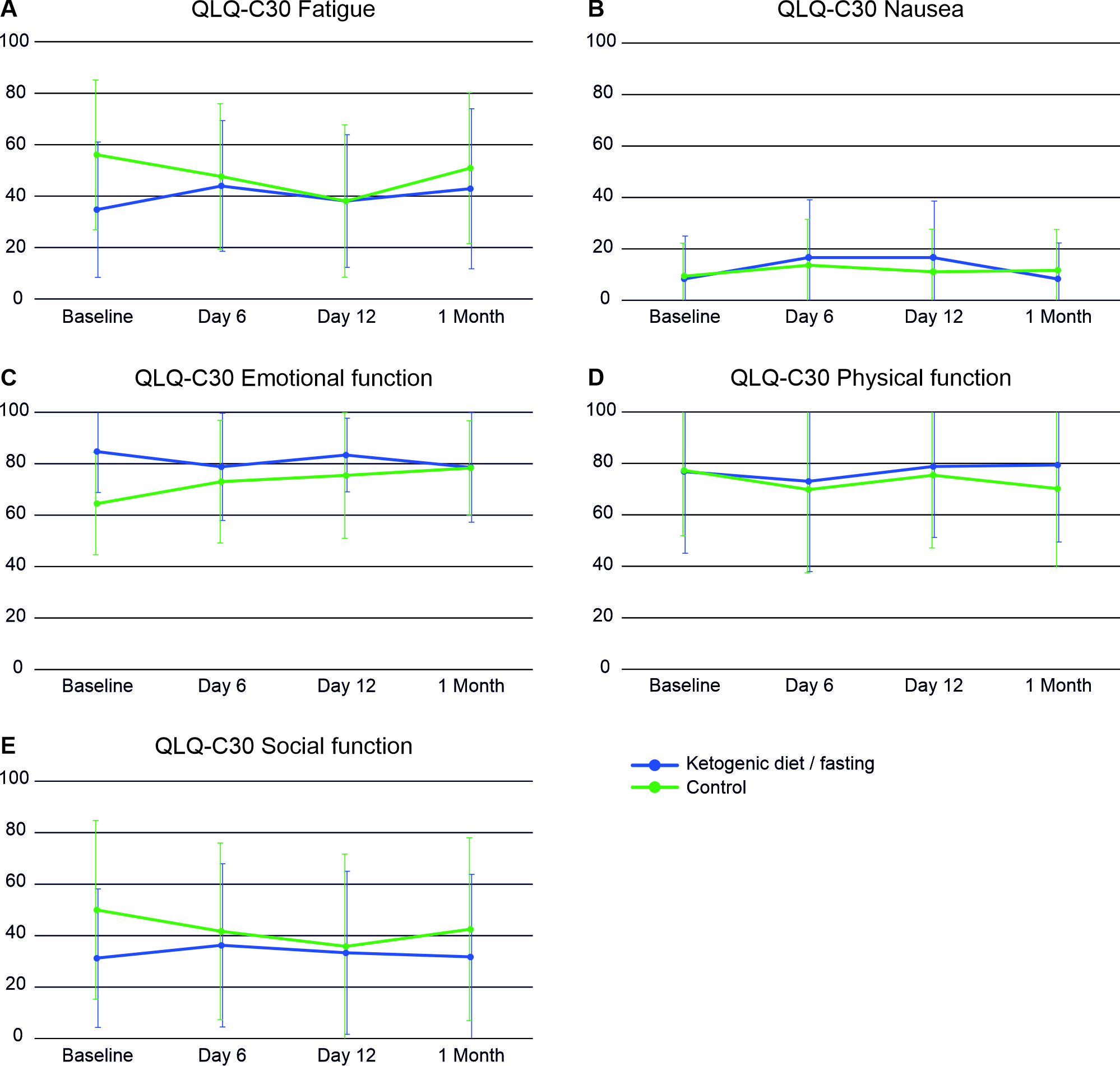


Aggregated scores of EORTC QLQ-C30. Mean values with standard deviation are indicated. For (A) and (B) a high score represents a high symptom burden while for (C-E), a high score represents a high functional level.

**Supplement Figure 3: Progression free survival by Body Mass Index**


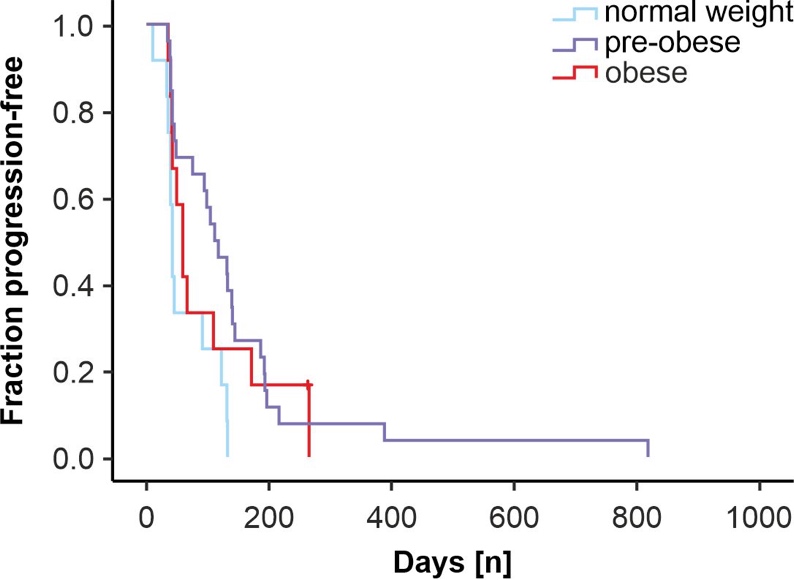


Analysis of the intention to treat population (n = 50) revealed the shortest progression free survival for the normal weight patients of mean 63 days (95%CI 38.9 – 87.8) compared to overweight patients with mean 146 days (95%CI 85.3–207.0, p =0.004) and obese patients with 100 days (95%CI 51–148.9, p =0.162).

**Supplement Figure 4: Weight loss**


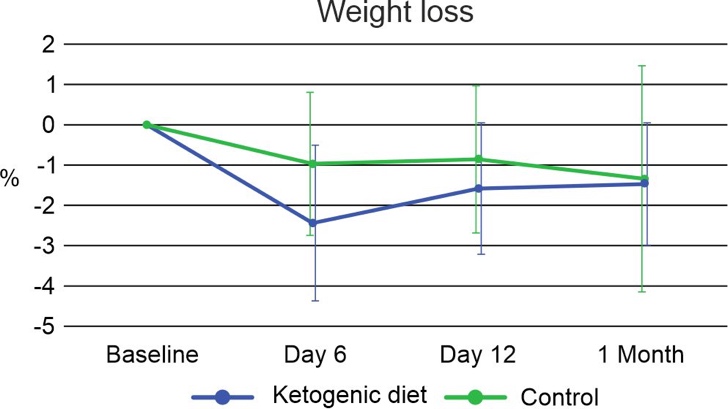


In the per protocol treated cohort mean weight at baseline was 85.7±11.9kg for KD-IF and 86.6±13.9kg for SD. Patients in the KD-IF group treated by protocol lost a median of 2.2% (mean absolute weight loss -2.2±1.9kg) of their body weight until day 6 in contrast to the SD group which lost a median of 0.7% (mean absolute weight loss -0.9±1.5kg) (*p* =0.014). The patients of the KD-IF group did not completely regain their weight with a median of -1.6% at day 12 and still -1.5% at the follow-up after one month.

**Supplement Table 1: Composition of Tavarlin ketogenic drink**

| **Average Content** | **per 100 ml** |
| --- | --- |
| Energy (kcal)  (kJ) | 181  748 |
| Fat (g)  - of which saturated fatty acids  - of which monounsaturated fatty acids  - of which polyunsaturated fatty acids | 14.4  2.4  5.7  6.4 |
| Carbohydrates (g)  - of which sugar | 1,2  0,5 |
| Fiber (g) | 2.8 |
| Protein (g) | 8.9 |

Ingredients: Water, rapeseed oil, pea protein, fiber: corn dextrin, medium-chain triglycerides (MCT), calcium phosphate, magnesium hydroxide, chromium chloride, iron gluconate, potassium chloride, potassium citrate, potassium iodate, potassium lactate, copper gluconate, manganese sulfate, Sodium selenate, zinc gluconate, sodium L-ascorbate, D-biotin, cyanocobalamin, menaquinone, nicotinamide, calcium-D-pantothenate, phylloquinone, pteroylglutamic acid, pyridoxine hydrochloride, retinyl acetate, riboflavin, DL-alpha-tocopheryl acetate, Cholecalciferol, vitamin E-rich oil from palm fruit / annatto, choline tartrate, L-carnitine, maltodextrin, aroma, emulsifier: mono- and diglycerides of fatty acids.

**Supplement table 2: Diet example of two patients during the days of calorie restricted ketogenic diet**

| **Day** | **Breakfast** | **Lunch** | **Dinner** | **Snack** | **Macronutrients** |
| --- | --- | --- | --- | --- | --- |
| 1 | ½ recipe 1: scrambled eggs with chives and bacon | 1 Tavarlin-Drink | ½ recipe 1: scrambled eggs with chives and bacon | - 1 Tavarlin-Drink - recipe 3: quark with raspberries | 23,7 kcal/kg/d  Protein: 21,6 % Fat: 70,5 % Carbohydrate: 10,3 % |
| 2 | ½ recipe 1: scrambled eggs with chives and bacon | - 1 Tavarlin-Drink  - ½ recipe 8: Chicken Curry | ½ recipe 8: Chicken Curry | 1 Tavarlin-Drink |  |
| 3 | ½ recipe 1: scrambled eggs with chives and bacon | - 1 Tavarlin-Drink  - ½ recipe 8: Chicken Curry | ½ recipe 8:  Chicken Curry | 1 Tavarlin-Drink |  |
| 7 | ½ recipe 1: scrambled eggs with chives and bacon | 1 Tavarlin-Drink | Recipe 7: Salad with chicken | 1 Tavarlin-Drink | 23,6 kcal/kg/d  Protein: 20,8 % Fat: 72,8 %  Carbohydrate: 8,9 % |
| 8 | ½ recipe 1: scrambled eggs with chives and bacon | 1 Tavarlin-Drink | Recipe 8: Chicken Curry | 1 Tavarlin-Drink |  |
| 9 | ½ recipe 1: scrambled eggs with chives and bacon | 1 Tavarlin-Drink | Recipe 7: Salad with chicken | 1 Tavarlin-Drink |  |
|  | | | | | |
| **Day** | **Breakfast** | **Lunch** | **Dinner** | **Snack** | **Macronutrients** |
| 1 | 1 Tavarlin-Drink | - 1 Tavarlin-Drink  - Recipe 1:  scrambled eggs with chives and bacon | 1 Tavarlin-Drink |  | 18,3 kcal/kg/d  Protein: 19 %  Fat: 75,3 %  Carbohydrate: 9,1 % |
| 2 | 1 Tavarlin-Drink | - 1 Tavarlin-Drink  - Recipe 1:  scrambled eggs with chives and bacon | 1 Tavarlin-Drink |  |  |
| 3 | 1 Tavarlin-Drink | - 1 Tavarlin-Drink  - Recipe 7: Salad with chicken | 1 Tavarlin-Drink |  |  |
| 7 | 1 Tavarlin-Drink | -1 Tavarlin-Drink  - recipe 6: Salad with cheese, olives and egg | 1 Tavarlin-Drink |  | 19,1 kcal/kg/d  Protein: 17,4 %  Fat: 78,1 %  Carbohydrate: 7,5 % |
| 8 | 1 Tavarlin-Drink | - 1 Tavarlin-Drink  - Recipe10:  Cauliflower Curry | 1 Tavarlin-Drink |  |  |
| 9 | 1 Tavarlin-Drink | - 1 Tavarlin-Drink  - Recipe 8: Chicken Curry | 1 Tavarlin-Drink |  |  |
